# Immunotherapeutic Targeting of Membrane Hsp70-Expressing Tumors Using Recombinant Human Granzyme B

**DOI:** 10.1371/journal.pone.0041341

**Published:** 2012-07-19

**Authors:** Mathias Gehrmann, Stefan Stangl, Andreas Kirschner, Gemma A. Foulds, Wolfgang Sievert, Brigitte T. Doß, Axel Walch, Alan G. Pockley, Gabriele Multhoff

**Affiliations:** 1 Department of Radiation Oncology, Klinikum rechts der Isar, Technische Universität München, and Clinical Cooperation Group (CCG) “Innate Immunity in Tumor Biology”, Helmholtz Zentrum München, Deutsches Forschungszentrum für Gesundheit und Umwelt, Munich, Germany; 2 Department of Oncology, The Medical School, University of Sheffield, Sheffield, United Kingdom; 3 Institute of Pathology, Helmholtz Zentrum München, Deutsches Forschungszentrum für Gesundheit und Umwelt, Munich, Germany; University Hospital of Heidelberg, Germany

## Abstract

**Background:**

We have previously reported that human recombinant granzyme B (grB) mediates apoptosis in membrane heat shock protein 70 (Hsp70)-positive tumor cells in a perforin-independent manner.

**Methodology/Principal Findings:**

Optical imaging of uptake kinetics revealed co-localization of grB with recycling endosomes (Rab9/11) as early as 5 min after internalization, with late endosomes (Rab7) after 30 min, and the lysosomal compartment (LAMP1/2) after 60 to 120 min. Active caspase-3-mediated apoptosis was induced in mouse CT26 monolayer cells and 3D tumor spheroids, but not in normal mouse endothelial cells. Granzyme B selectively reduced the proportion of membrane Hsp70-positive cells in CT26 tumor spheroids. Consecutive i.v. injections of recombinant human grB into mice bearing membrane Hsp70-positive CT26 tumors resulted in significant tumor suppression, and a detailed inspection of normal mouse organs revealed that the administration of anti-tumoral concentrations of grB elicited no clinicopathological changes.

**Conclusions/Significance:**

These findings support the future clinical evaluation of human grB as a potential adjuvant therapeutic agent, especially for treating immunosuppressed patients that bear membrane Hsp70-positive tumors.

## Introduction

Heat shock protein 70 (Hsp70) is frequently overexpressed in tumors and cytosolic Hsp70 mediates the protection of tumor cells against environmental stress [1]–[3]. Hsp70 has also been found to be localized in the plasma membrane of a large proportion of different tumor entities, but not in the plasma membrane of normal cells/tissues [4]–[11]. Although the precise role of membrane-associated Hsp70 is not fully understood, overall survival of patients with lower rectal carcinomas and non-small cell lung cancer (NSCLC) exhibiting a membrane Hsp70-positive phenotype has been found to be significantly lower than that of their membrane Hsp70-negative counterparts [12]. Furthermore, most standard therapies, including radiochemotherapy, increase the membrane densities of Hsp70 on cancer, but not normal cells [7], [8], [13]. These findings highlight the clinical significance of determining the membrane Hsp70 status, and the urgent need for innovative treatment modalities that can specifically target highly aggressive, membrane Hsp70-positive tumors.

We have previously demonstrated that membrane Hsp70 serves as a tumor-specific recognition structure for pre-activated natural killer (NK) cells, but not for resting NK cells [14]. Full-length Hsp70, as well as the extracellularly-accessible Hsp70-derived peptide TKDNNLLGRFELSG (TKD), in combination with low dose IL-2 increase the expression density of activating receptors such as NKG2D, NKG2C/CD94 and NCRs and stimulate the cytolytic activity of NK cells to attack membrane Hsp70-positive tumor cells *in vitro* [5], [14]. Tolerability and safety of patient-derived, *ex vivo* TKD/IL-2-activated NK cells as an immunotherapeutic option has been demonstrated in a Phase I clinical trial [15], [16] and a proof-of-concept Phase II study in NSCLC patients following radiochemotherapy is ongoing.

The mechanism by which activated NK cells kill membrane Hsp70-positive tumor cells is associated with an enhanced production and release of the pro-apoptotic serine protease Granzyme B (grB) [17]. Sepharose column chromatography has revealed that the epitope of Hsp70 which is exposed to the extracellular milieu on tumor cells enables binding of recombinant human grB [17]. Furthermore, we have demonstrated that grB-induced apoptosis in Hsp70-positive tumor cells occurs in the absence of perforin [17], and that the interaction of grB with the membrane form of Hsp70 is dependent on an eukaryotic glycosylation pattern of grB [18]. It has also been shown that membrane Hsp70 shows a fast turn-over rate [19] and this might enable the uptake of grB.

Presuming that grB is only internalized into membrane Hsp70-positive tumor cells, but not in healthy tissues that lack membrane Hsp70, human grB might provide a novel strategy to induce tumor cell apoptosis in a highly selective manner with a low risk of generating adverse effects. This study therefore investigates the potential of the therapeutic potential of grB using 3D tumor spheroids and a syngeneic CT26 tumor mouse model. The internalization pathway into tumor cells has been visualized using fluorophor-conjugated grB and confocal microscopy.

Our findings demonstrate that grB selectively induces caspase-3 dependent apoptosis in membrane Hsp70-positive cells in CT26 mouse tumor cell monolayers and spheroids. Furthermore, the administration of grB significantly reduces the size of solid tumors in mice. The lack of any adverse effects in mice receiving 4 repeated injections of grB supports the proposition that grB might be effective for the treatment of tumor patients that lack active immune protection during and/or directly after therapeutic interventions such as radiochemotherapy.

## Results

**Figure 1:**
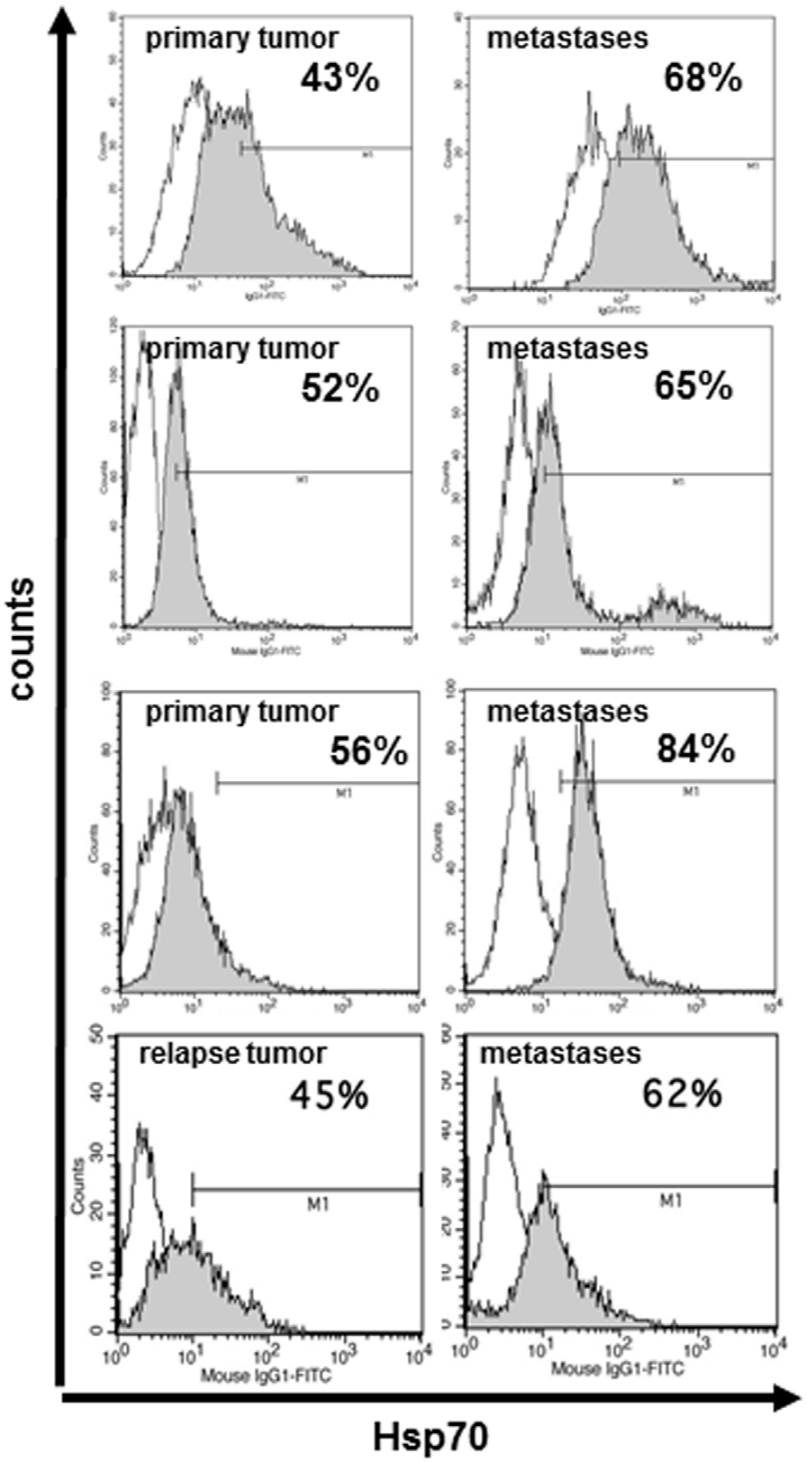
Comparative flow cytometric histograms of membrane Hsp70 expression on viable (7-AAD negative) cells from primary tumors and distant metastases of three patients, and on a relapse tumor and a distant metastases of another patient using FITC-labelled IgG1 isotype-matched control antibody (open histogram) and cmHsp70.1 mAb (grey histogram). The mean fluorescence intensity of Hsp70 is much higher on metastases compared to primary and relapse tumors, as indicated by a shift of the grey peak to the right.

In contrast to normal cells, tumors frequently express Hsp70 on their plasma membrane [4], and we show here that the membrane density of Hsp70 is considerably higher on metastases compared to primary and relapse tumors (**Fig. 1**). As grB has previously been shown to selectively initiate perforin-independent apoptosis in membrane Hsp70-positive human tumor cells [17], herein we studied the capacity of HEK293 cell-derived, recombinant human grB [18] to kill CT26 mouse colon adenocarcinoma cells. Approximately 60% of the cells in monolayer and 3D tumor spheroids express Hsp70 on their plasma membrane.

**Figure 2:**
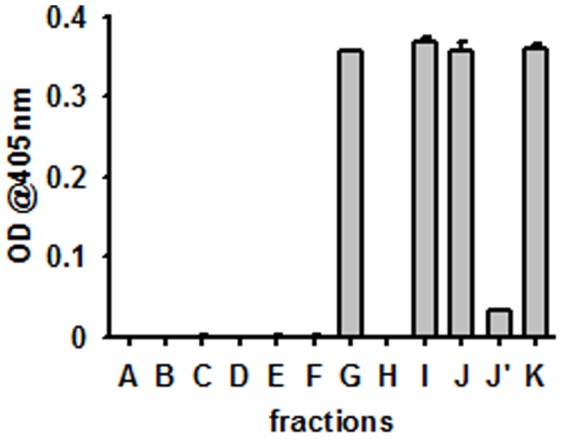
Enzymatic activity during the purification of grB. **A**–**F** grB before enterokinase digestion; H, J’ flow through during purification process; G, I, J, K grB after enterokinase digestion: **A.** cell culture supernatant of the HEK293 producing cells; **B.** after addition of imidazole; **C.** flow through of Ni column; **D.** His-tagged grB; **E.** pooled His-tagged grB; **F.** after exchange to enterokinase buffer; **G.** after enterokinase digestion; **H.** flow through of heparin column; **I.** pooled grB containing fractions after heparin column; **J.** concentrated grB; **J’.** flow through from concentration procedure; **K.** sterile filtered (0.2 µl) active grB (final product).

Active and inactive, HEK293 cell-derived, recombinant human grB was generated as described previously [18] and its enzymatic activity at different stages in the process was monitored (**Fig. 2**). The endotoxin content of grB preparations before and after enterokinase digestion was determined using the E-TOXATE Kit (Sigma-Aldrich, Catalog #ET0100) and was found to be below the detection limit of the assay (0.05–0.1 endotoxin units (EU)/mL) in each fraction (data not shown).

### Induction of Apoptosis in CT26 Mouse Tumor Cell Monolayers by Human Granzyme B

**Figure 3:**
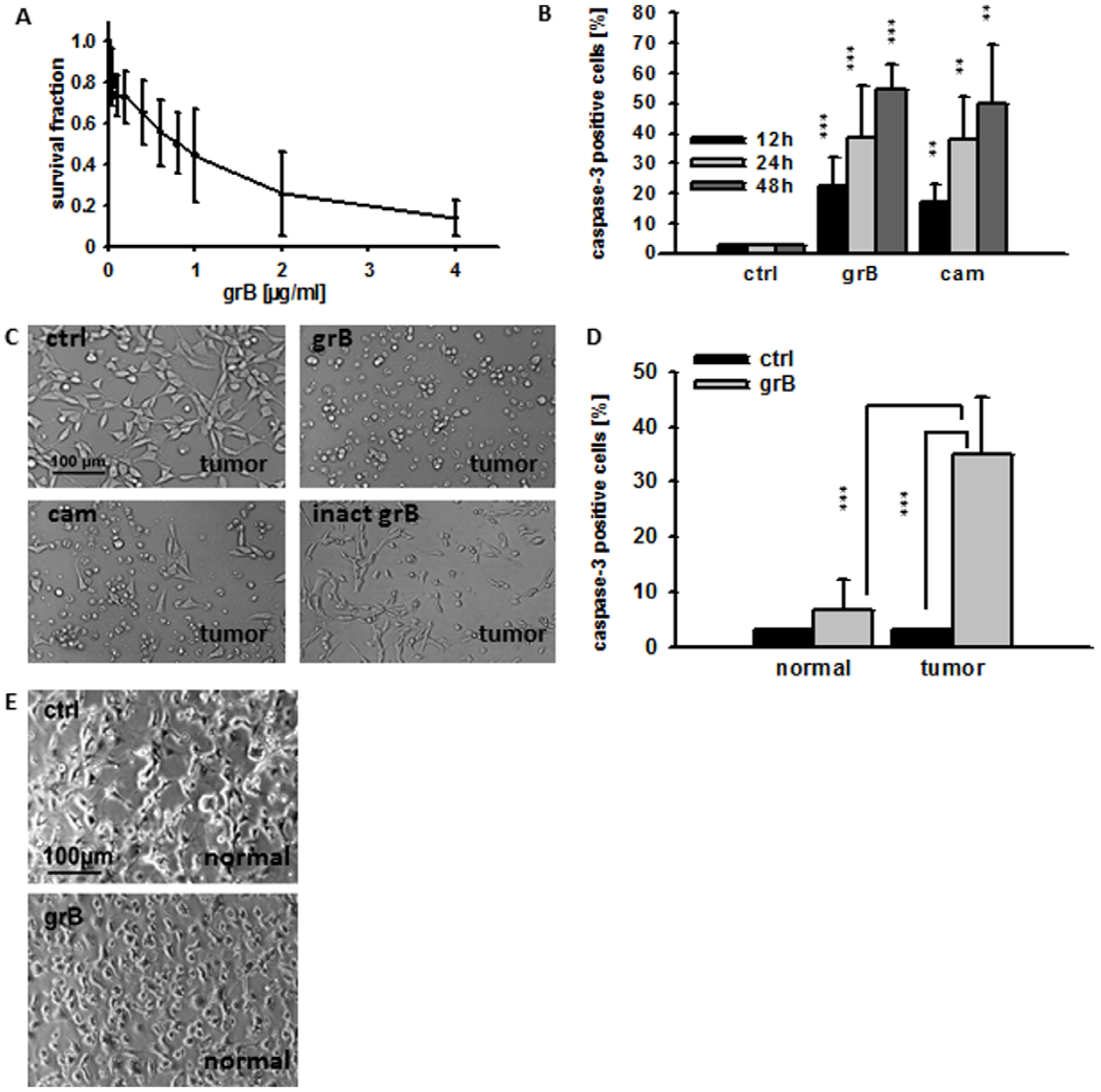
Recombinant human grB induces apoptosis in monolayer CT26 tumor, but not normal cells. (**A**) Clonogenic cell survival of CT26 tumor cells after treatment with active human grB (0.04, 0.2, 0.4, 0.6, 0.8, 1, 2, 4 µg/ml) on day 6. The data represent the mean of three independent experiments ± S.D. Significant differences in the survival of grB-treated CT26 vs. untreated controls were seen from 0.6 µg/ml upwards (p*<0.05). (**B**) CT26 cells were treated with PBS (ctrl), granzyme B (4 µg/ml; grB) or camptothecin (4 µg/ml; cam) for 12 h (black bars), 24 h (grey bars), and 48 h (white bars). Permeabilized cells were stained with a FITC-conjugated active caspase-3 specific antibody and the percentage of active caspase-3 positive cells determined by flow cytometry. The data represent the mean of 3–10 independent experiments ± S.D. (p***<0.001; p** = 0.01). (**C**) Light microscopical views of CT26 tumor cells after treatment with PBS (ctrl), grB (4 µg/ml), cam (4 µg/ml) or inactive grB (inact grB) for 48 h (20x objective, scale bar 100 µm). Representative images are shown from at least 3 experiments using CT26 tumor cells (**D**) CD31-positive mouse endothelial cells from healthy BALB/c mice (normal) and CT26 tumor cells (tumor) were treated either with PBS (ctrl; black bars) or grB (4 µg/ml; grey bars) and the percentage of cells that positively stained for active caspase-3 was determined by flow cytometry. The data represent the mean of six independent experiments ± S.D. Asterisks represent significantly different values (p*** = 0.001). Inactive grB showed similar results like PBS (data not shown) (**E**) Light microscopic phase contrast analyses of adherent growing CD31-positive mouse endothelial cells (normal) treated for 24 h with PBS (ctrl, upper graph) or grB (4 µg/ml). Both untreated and treated CD31-positive mouse endothelial cells show regular cell morphology. Similar results were obtained in three independent experiments (20x objective, scale bar 100 µm).

The enzymatic activity of grB in conditioned medium containing tumor cells did not differ significantly between day 0 to day 3 (data not shown). GrB significantly reduced the clonogenic survival of CT26 tumor cells at concentrations of 0.6, 0.8, 1, 2, and 4 µg/ml (**Fig. 3A**). Apoptosis (on the basis of active caspase-3 expression) was determined 12, 24 and 48 h after incubation of CT26 monolayer cells with grB (4 µg/ml). The percentage of caspase-3 positive cells increased from 3±0% (PBS control) to 22±9% (12 h, black bars), to 39±17% (24 h, light grey bars) and to 54±8% (48 h, dark grey bars) after different times of grB treatment (**Fig. 3B**). Similar effects were induced by the topoisomerase inhibitor camptothecin (cam) which was used as a positive control (**Fig. 3B**). Light microscopy performed before and 48 h after treatment with grB (4 µg/ml) and cam (4 µg/ml) confirmed apoptosis in adherent CT26 tumor cells on the basis of reduced adherence, cell rounding and an increase in granularity. In contrast, inactive grB exhibited no effects (**Fig. 3C**).

Given that the therapeutic application of grB in an *in vivo* setting will require intravenous injection, it is important to ascertain the potential effects of grB on endothelial cells (ECs). The effects of grB on the viability of CD31-positive ECs that were freshly isolated from normal BALB/c mice were therefore assessed. As shown in **Fig. 3D**, the percentage of caspase-3 positive mouse ECs remained unchanged after treatment with grB (4 µg/ml; 3±0% to 7±6%; p = 0.2) and normal mouse ECs retained their adherence and regular morphology (**Fig. 3E**).

### Internalization of Granzyme B

**Figure 4:**
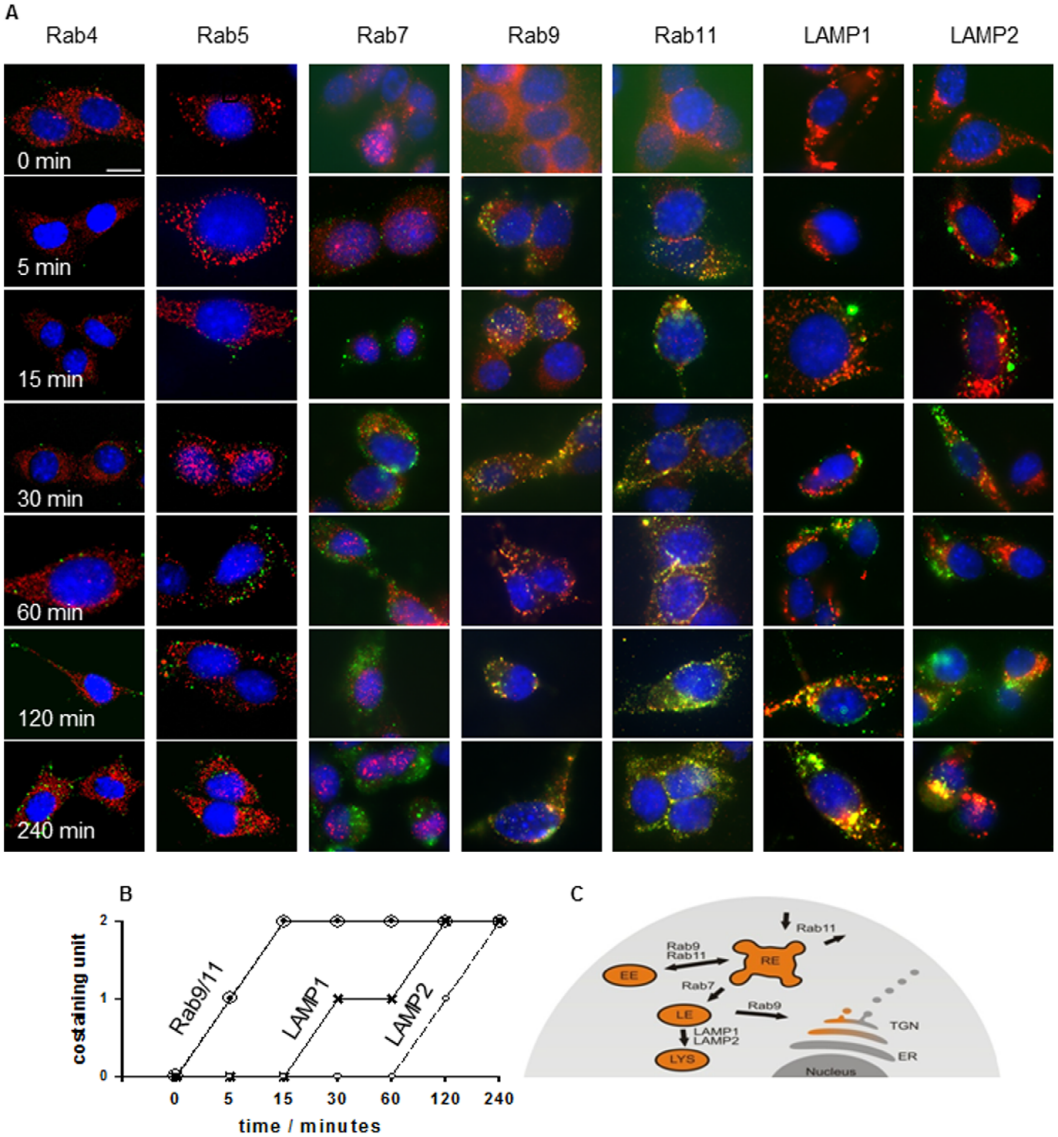
Confocal analysis of immunofluorescence staining of grB (ATTO488, green) and Rab4, Rab5, Rab7, Rab9, Rab11, LAMP1, and LAMP2 (Cy3, red). (**A**) CT26 tumor cells were grown on MatTek Glass Bottom Culture Dishes then incubated with 4 µg/ml ATTO488-labeled grB for 0, 5, 15, 30, 60, 120 and 240 min. After fixation, cells were stained with anti-endosomal (Rab4, Rab5, Rab7, Rab9, Rab11) or lysosomal antibodies (LAMP1, LAMP2) followed by appropriate Cy3-labeled secondary antibodies. Cells were mounted in DAPI-Vectashield and imaged using a Zeiss LSM 510 Inverted microscope (63x objective). Co-localization, as determined by an overlap of the green and red fluorescence, is visible as yellow. One representative image from 2 independent experiments is shown (scale bar, 10 µm). (**B**) Semi-quantitative analysis of the co-staining intensity and kinetics of endocytosis of grB and Rab9/11 and LAMP1/LAMP2. (**C**) Schematic representation of the mode of uptake and intracellular trafficking of grB in tumor cells.

The uptake of grB into membrane Hsp70-positive tumor cells was visualized using confocal microscopy. As illustrated in **Fig. 4A** **,** grB co-locates with recycling endosomes (Rab9/Rab11) as early as 5 min after endocytosis, and with late endosomes (Rab7) after 30 min. GrB subsequently resides in lysosomal compartments 60 to 120 min after internalization, as visualized by co-staining with fluorophor-labeled LAMP1 and LAMP2 antibodies (**Fig. 4A**). The kinetics and tumor-specific internalization of grB are schematically represented in **Fig. 4B** and **Fig. 4C** respectively.

### Induction of Apoptosis in CT26 Mouse Tumor Spheroids by Human Granzyme B

**Figure 5:**
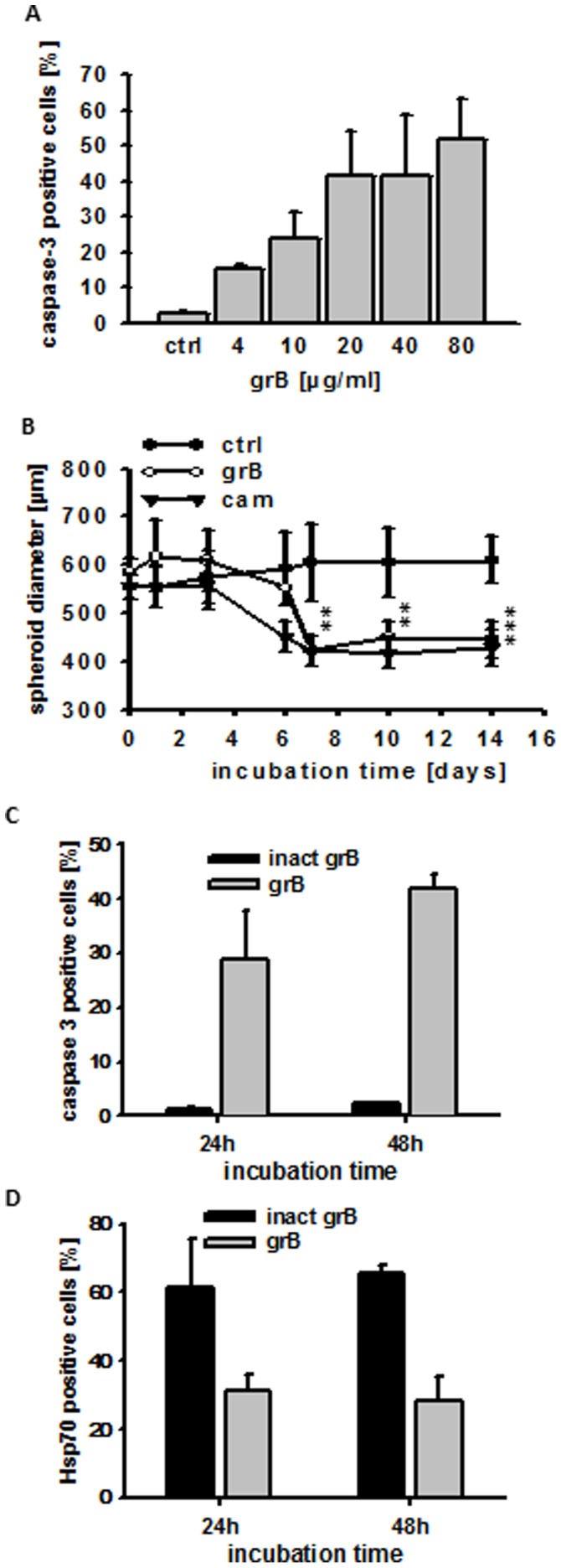
Recombinant human grB induces apoptosis in CT26 tumor spheroids. (**A**) CT26 tumor spheroids were treated either with PBS (ctrl) or grB (4, 10, 20, 40, 80 µg/ml) for 48 h. Single cell suspensions were generated from tumor spheroids by trypsinization and the cells permeabilized before being stained for active caspase-3. The percentage of active caspase-3 positive cells was determined using a BD Biosciences FACSCalibur™. The data represent mean values of 3–4 independent experiments ± S.D. All grB-treated samples significantly differed from ctrl values (p***<0.001). (**B**) CT26 tumor spheroids were incubated for up to 14 days either with PBS (ctrl; filled circles), grB (80 µg/ml; open circles) or cam (4 µg/ml; filled triangles). Diameters of spheroids were measured on days 0, 1, 3, 6, 7, 10, and 14 after treatment. The data represent mean values of 6 independent experiments ± S.D. After a slight increase in diameter size, grB initiated a significant shrinkage of spheroids from day 7 onwards (day 7, p** = 0.004; day 10, p** = 0.0063; day 14, p*** = 0.0003). A significant reduction in tumor size was observed after treatment with cam from day 6 onwards (day 6, p** = 0.0061; day 7, p** = 0.0022; day 10, p** = 0.0019; day 14, p** = 0.0012). (**C**) Effect of treatment with inactive and active grB for 24 h and 48 h on the percentage of active caspase-3 positive cells in CT26 cells isolated from tumor spheroids. (**D**) Effect of treatment with inactive and active grB for 24 h and 48 h on the percentage of membrane Hsp70-positive CT26 cells in tumor spheroids. Spheroids were incubated with activated or inactivated grB for the indicated time periods, after which single cell suspensions were prepared following trypsinization. Cells were then incubated with cmHsp70.1-FITC mAb and membrane Hsp70 expression was determined by flow cytometry.

The approximate amount of grB that might be needed to effectively target tumors *in vivo* was assessed by measuring the capacity of grB to induce apoptosis in 3D tumor spheroids on day 2 after incubation with 4, 10, 20, 40 and 80 µg/ml grB. Concentrations of grB as low as 4 µg/ml significantly increased the proportion of caspase-3 positive cells (3±2% to 8±0%), and this effect gradually increased at higher grB concentrations (57±3% caspase-3 positive cells at 80 µg/ml) (**Fig. 5A**). No significant increase in apoptosis was observed for PBS-treated spheroids (ctrl).

Morphological changes in CT26 tumor spheroids after co-incubation with grB or cam were determined, and their diameters were measured. Untreated spheroids are round with a clearly defined, smooth border. Tumor spheroids exposed to grB lost their integrity and regular shape but significant shrinkage was only observed from day 7 onwards. In contrast, spheroids treated with cam (4 µg/ml) remained regular during shrinkage (data not shown). A slight initial swelling of tumor spheroids was apparent between 1 and 3 days after treatment with grB (**Fig. 5B**). This is most likely due to loss of integrity which is induced by apoptotic cell death, as is illustrated by the caspase-3 apoptosis assay (**Fig. 5A**).

The overall size of spheroids that had been treated with grB and cam was significantly decreased on day 14 (583±30 µm to 446±36 and 556±25 µm to 429±37 µm respectively (**Fig. 5B**), whereas the size of untreated, control spheroids had increased (555±27 µm to 610±48 µm, **Fig. 5B**). The initial sizes of the spheroids were similar across the treatment groups (565±30 µm). These findings demonstrate that grB induces apoptosis in membrane Hsp70-positive cells in monolayers and 3D tumor spheroids.

Caspase-3 positivity was only observed in tumor spheroids that had been treated with active grB (**Fig. 5C**). The specificity of grB-mediated killing was demonstrated by the finding that the percentage of membrane Hsp70-positive cells in CT26 tumor spheroids that had been treated with active grB reduced from 60% to 40%. In contrast, the treatment of CT26 tumor spheroids with inactive grB had no effect on the percentage of membrane Hsp70-positive cells contained therein (**Fig. 5D**).

### GrB Induces Tumor Suppression and is Well Tolerated in Tumor-bearing Mice

**Figure 6:**
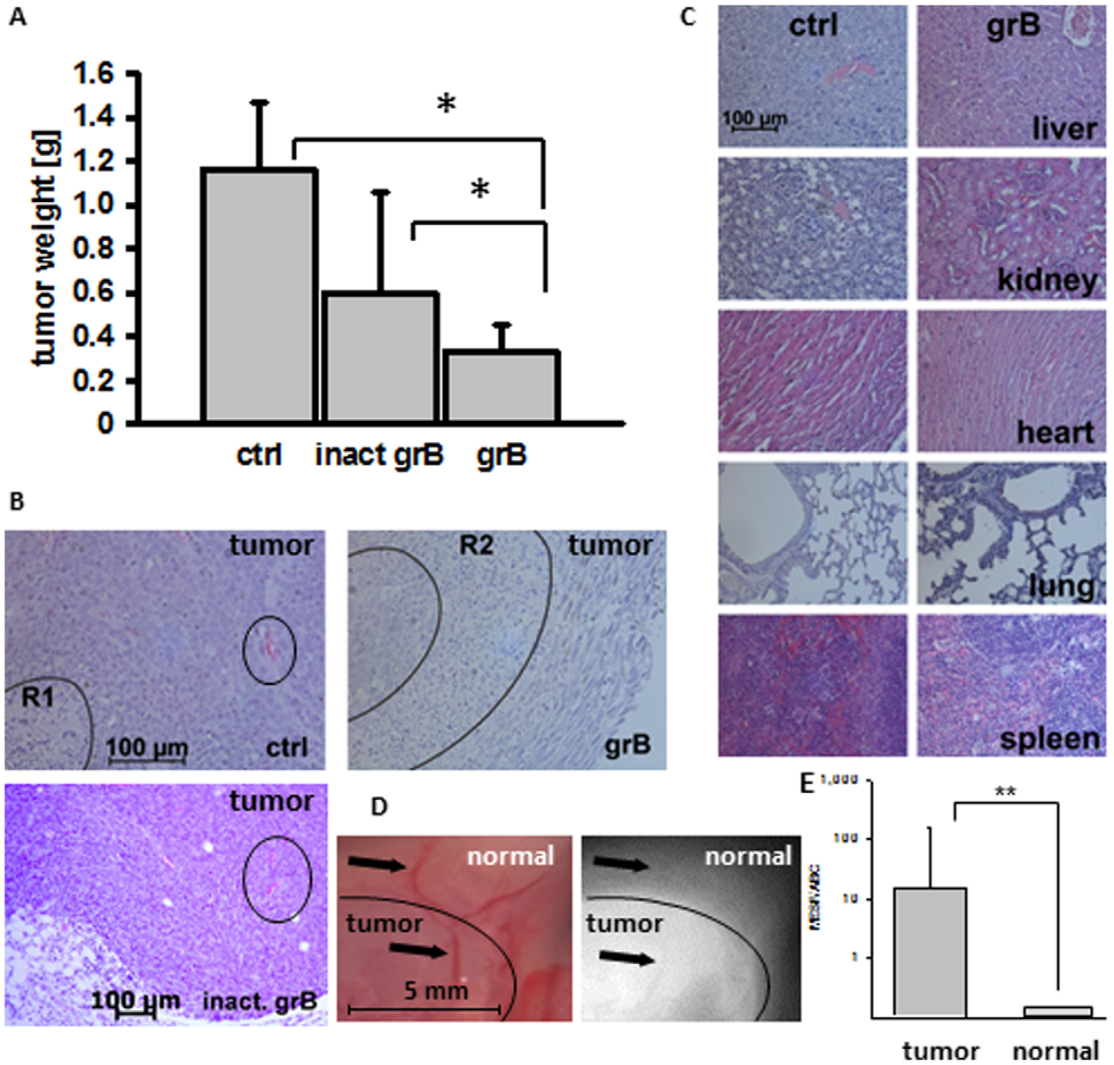
Active human grB results in tumor regression without the induction of adverse effects. (**A**) CT26 tumor-bearing BALB/c mice were injected (i.p.) either with PBS (ctrl), inactive grB (inact grB) or enzymatically active grB (grB; 20 µg/g mouse body weight in 200 µl PBS) on days 6, 7, 13, 14 after i.p. injection of CT26 tumor spheroids. Mice were sacrificed on day 21. The weights of tumors in mice treated with grB were significantly lower than those in the control group (n = 15 per group; p*<0.05). The weight of tumors in mice treated with active or inactive grB were also significantly different (p*<0.05), whereas there was no significant difference in the weights of tumors in control mice or mice that had been treated with inactive grB (*p* = 0.49). (**B**) Comparison of CT26 tumors derived from control mice (ctrl), active and inactive grB-treated mice (see above). Representative sections of formalin-fixed paraffin-embedded tumors stained with H&E. R1 marks necrotic/apoptotic areas in the centre of the tumor of ctrl animals; R2 marks necrotic/apoptotic areas in the border area of the tumor of active grB-treated mice; vessels are marked with a circle. (**C**) Comparison of sections from formalin-fixed paraffin-embedded liver, kidney, heart, lung and spleen derived from control mice and mice treated with grB (see above) stained with H&E were analyzed using light microscopy (20x objective, indicated scale bar 100 µm). (**D**) Intraoperative detection of Cy5.5-labeled cmHsp70.1 mAb in BALB/c mice bearing CT26 tumors. Antibody (100 µg) was injected (i.v.) into the tail vein on day 6 after tumor injection. Representative false color images and views of the Cy5.5 fluorescence of the neck part of three mice were taken 24 hours after i.v. injection. The circled area indicates the histologically proven tumor tissue; arrows indicate superficial blood vessels in the normal tissue (upper arrow) and the tumor (lower arrow). (**E**) Relative binding of cmHsp70.1 mAb to isolated CT26 tumor cells (tumor) and endothelial (normal) cells. The capacity of cells to bind cmHsp70.1-FITC is presented as mean equivalents of soluble fluorochrome (MESF) divided by the antibody binding capacity (ABC) and was determined using the Quantum Simply Cellular anti-Mouse IgG according to the manufacturer’s protocol (Bangs Laboratories, Inc., Fishers, IN, USA). The Y-axis is a logarithmic scale.

In order to determine the anti-tumoral activity of human grB *in vivo,* mice were injected (i.p.) with CT26 tumor spheroids of identical size. Tumor-bearing mice were injected (i.v.) with PBS (200 µl; ctrl) or with 20 µg/g body weight inactive or active grB diluted in 200 µl PBS on days 6, 7, 13, and 14. Mice were sacrificed on day 21, at which time tumors and organs (liver, kidney, heart, lung, spleen) were collected. A significant reduction in tumor weight was observed only in mice treated with active grB (grB; 0.33±0.12 g; p* = 0.04 vs. 1.17±0.31 g in control animals) (**Fig. 6A**). Treatment of mice with inactive grB preparations resulted in a non-significant reduction of the tumor weight (inact grB; 0.60±0.45 g; *p* = 0.49) compared to control tumors (**Fig. 6A**). Overall survival of mice that had been treated with active human grB was significantly greater than that of control mice (p* = 0.023; data not shown).

Four tumors were analysed by H&E staining. Viable tumor tissue is characterized by numerous areas of mitosis, compact tissue, and rare single cell apoptosis. In contrast, necrotic/apoptotic areas are characterized by an increased eosin staining, the presence of apoptotic bodies, bulked extracellular matrices, dense nuclei and nuclear fragmentations. A representative view of one of the sections is shown in **Fig. 6B**. In all control animals carrying larger tumors than grB-treated animals (n = 4), necrosis was only found in the central part (marked with R1). In contrast, necrosis was predominantly found on the outer borders (marked with R2), and in proximity to blood vessels (circled) in tumor-bearing mice that had been treated with active grB. This result was also confirmed in 4 out of 4 animals. None of the tumors treated with active grB exhibited central necrosis. Tumors in mice treated with inactive grB showed no reduction in size, and no damage to tumor tissue in the vicinity of vessels was apparent (**Fig. 6B** **,** vessels are circled).

Although no obvious adverse side-effects occurred following the administration of active human grB, the organs of control mice and of those treated with active grB exhibited no pathophysiological changes (**Fig. 6C**). Individual organs exhibited normal tissue architecture, with no evidence of infarcts or internal bleeding. Active human grB at concentrations that can reduce tumor size does therefore not induce detectable histological or morphological changes in normal organs.

The Hsp70 status of tumor and normal tissues in CT26 tumor-bearing mice was determined by measuring the *in vivo* binding capacity of fluorescently-labelled cmHsp70.1 antibody. For this, intraoperative *in vivo* imaging of tumor-bearing mice was performed 24 h after tail vein injection of 100 µl of a 1 mg/ml dilution of Cy5.5-labelled cmHsp70.1 mAb in PBS. The fluorescence of the dissected tumor and of the surrounding normal tissue was measured and compared to the relevant normal tissue by false color imaging. A representative fluorescent image revealed an accumulation of cmHsp70.1 mAb in the tumor but not in the adjacent normal tissue and in the blood vessels (**Fig. 6D**). The delineation of tumor and normal tissue was confirmed using H&E staining of the imaged area (data not shown).

The relative binding of cmHsp70.1 mAb to isolated tumor and endothelial cells was determined by flow cytometry using Quantum Simply Cellular beads. This revealed a significantly higher binding of cmHsp70.1 mAb to tumor cells compared to endothelial cells (**Fig. 6E**).

## Discussion

Granzyme B (grB) derived from *E. coli* [20] has been shown to induce apoptosis in tumor target cells via perforin pores that trigger the release of endocytosed grB [21]. Lytic agents such as cationic lipid formulation Bioporter® (Gene Therapy Systems Inc., San Diego, CA, USA) can be used to facilitate the release of grB [22], [23]. The activity of grB purified from cytotoxic T and NK cells has been found to be weak due to a covalent binding of grB to the human protease inhibitor PI-9 [24]–[26]. Furthermore, Trapani and co-workers [28] have shown that more than 50% of total grB resides in the nucleus and not in the cytosol of human effector cells.

We have described a perforin-independent pathway by which human grB can specifically induce apoptosis in membrane Hsp70-positive tumor cells [17]. Mammalian glycosylation and posttranslational modifications are a prerequisite for this Hsp70-mediated uptake of human grB into tumor cells [18], since not even *Pichia pastoris* derived grB [27] was effective. The present study used a mammalian HEK293 expression system to produce high yields of enzymatically and biologically active, glycosylated human grB following enterokinase (EK) cleavage of the inactivation site [18].

In the presence of perforin, grB-mediated apoptosis in monolayer cells is typically induced after an incubation period of 4 h to 48 h at concentrations ranging between 10 ng/ml and 10 µg/ml [20], [24]. Herein, we show that human grB induces apoptosis in membrane Hsp70-positive mouse tumor cells, but not in normal mouse tissues in a comparable time and concentration range.

Dose-response effects, as determined by measuring clonogenic cell survival assays, revealed a significant reduction in clonogenicity already at a very low grB concentration (0.6 µg/ml). Less than 20% of the tumor colonies were found to be alive at a concentration of 4 µg/ml of human grB. In order to estimate the concentration of human grB that might be necessary to treat solid mouse tumors, multicellular 3D tumor spheroid assays were performed. Tumor spheroids mimic the cellular and organotypic histomorphological features of tumors without vascularization [29]–[32]. In comparison to monolayer cells, a 20-fold higher concentration of human grB was required to induce the shrinkage of tumor spheroids. In contrast, significant active caspase-3 positivity could be achieved in tumor spheroids at grB concentrations as low as 4 µg/ml. These findings indicate that spheroid diameter is not an indicative surrogate for grB-mediated tumor cell killing. Cell death and loss in spheroid integrity following treatment with grB was also associated with irregularly shaped spheroid surfaces. In contrast, treatment with cam resulted in a gradual decrease in spheroid size. GrB predominantly induces apoptosis through mitochondrial leakage and activation of the caspase cascade [33]. In contrast, cam does not significantly interfere with the mitochondrial apoptosis pathway [34], but causes apoptosis via inhibition of the DNA topoisomerase I. Despite these mechanistic differences, grB and cam showed similar results with respect to the induction of active caspase-3-mediated apoptosis in membrane Hsp70-positive tumor cells [18].

Although mouse and human grB show distinct structural and functional characteristics, and despite slight differences in their substrate specificity, we have shown that human grB effectively initiates cell death in membrane Hsp70-positive mouse tumor cells. *In* *vitro* experiments indicated that procaspase-3 is identically cleaved by human and mouse-derived grB [35]. However, differences exist with respect to the cleavage of Bid by human and mouse grB. Bid, the cleavage product which results in the release of cytochrome c from mitochondria, is not processed as efficiently by mouse grB, as it is by human grB [34], [36]–[38]. Regarding these differences, it is assumed that doses which are effective in mice might have to be altered and adapted to the human system.

Our mouse data reveal that 4 consecutive treatment cycles using 20 µg grB per g body weight significantly reduce tumor weight, and the safety and tolerability of the treatment was proven by immunohistochemistry. No pathophysiological changes were found in the liver, kidney, lung, heart and spleen of the mice after this treatment regimen. The concentrations that were used in our study are relatively low compared to those used by Rosenblum and Liu who injected 5×37.5 mg per g body weight *E. coli* immunotoxin coupled to grB without inducing any severe side effects [22], [23]. Upcoming dose-escalation experiments will determine the maximal effective and tolerated dose.

Serum levels of active grB in healthy human individuals range between 15 and 40 pg/ml and can exceed up to 250 pg/ml during severe infections [39], [40]. Our study therefore used grB concentrations that are similar to those that have previously been used to induce perforin-dependent apoptosis *in vitro,* but higher than naturally-occurring levels. However, no adverse effects were associated with the administration of active grB.

It was also interesting to observe that necrotic regions in the tumor tissue were selectively found in proximity to tumor blood vessels and in peripheral tumor areas after an i.v. injection of active grB into mice, whereas necrosis was only seen in central areas of the tumors in control animals with larger tumor volumes. These data suggest that grB targets tumors via blood vessels.

On the basis of our findings, we propose that patients during and/or after standard radiochemotherapy might especially profit from receiving biologically active human grB. Our results warrant future clinical testing of human grB as a potential adjuvant therapeutic agent for the treatment of transiently immunosuppressed tumor patients with a membrane Hsp70-positive tumor phenotype.

## Materials and Methods

All chemicals and reagents were obtained from Roth (Carl Roth GmbH & Co, KG, Karlsruhe, Germany) or Sigma-Aldrich (Sigma-Aldrich, Inc., St. Louis, MO, USA) unless stated otherwise.

### Cells and Cell Culture

Human tumor and metastases specimens were obtained from tumor patients at the Klinikum rechts der Isar, TU München. Fresh biopsy material was washed in antibiotic-(penicillin/streptomycin) containing RPMI 1640 medium and single cell suspensions were prepared by mincing the tissue and forcing it through a sterile mesh. The study was approved by the Institutional Review Board of the Medical Faculty of the Klinikum rechts der Isar, TU München, Germany and all patients included in the study provided signed informed consent. All clinical investigations have been deducted according to the principles expressed in the Declaration of Helsinki.

The mouse colon adenocarcinoma cell line CT26 (CT26.WT; ATCC CRL-2638) [41], which is derived from a BALB/c mouse tumor was cultured in RPMI 1640 medium containing 10% (v/v) heat-inactivated fetal calf serum (FCS), 2 mM L-glutamine, 1 mM sodium-pyruvate, antibiotics (100 IU/ml penicillin and 100 µg/ml streptomycin) at 37°C in 5% (v/v) CO_2_. This cell line is 60% positive for membrane-Hsp70 expression, as determined by flow cytometry [11]. Single-cell suspensions were derived by short-term (less than 1 min) treatment with 0.25% (w/v) Trypsin-0.53 mM EDTA.

CD31-positive endothelial cells (normal cells) were isolated from BALB/c mice using magnetic dynabeads (Invitrogen, Karlsruhe, Germany) coated with CD31 antibody (BD Biosciences; Heidelberg, Germany) according to the manufacturer’s instructions (Invitrogen). The cells were cultured in endothelial cell growth medium (PromoCell, Heidelberg, Germany) for up to 4 cell passages.

All cell lines were regularly screened for mycoplasma contamination using an enzyme immunoassay which detects *Mycoplasma arginini*, *Mycoplasma hyorhinis*, *Achdeoplasma laidlawii*, and *Mycoplasma orale* (Roche Diagnostics, Basel, Switzerland). Only mycoplasma-free cell lines were used in this study.

### Flow Cytometry

The membrane Hsp70 phenotype on viable tumor and metastatic cells of patients was determined by flow cytometry using FITC-conjugated cmHsp70.1 monoclonal antibody (mAb; multimmune GmbH, Germany) an IgG1 isotype-matched control antibody, as described elsewhere [11].

### Confocal Microscopy

CT26 cells were seeded into MatTek Glass Bottom Culture Dishes (1×10^5^ cells in 2 ml RPMI 1640 medium). After 48 h, culture medium was exchanged for RPMI 1640 medium containing grB (4 µg/ml) and incubated for various durations (0, 5, 15, 30 min, 1, 2, 4 h). Cells were then washed in PBS, fixed in 0.25% w/v paraformaldehyde, permeabilized in PBS containing 0.1% w/v saponin and 0.1% w/v BSA and stained with primary antibodies to Rab4, Rab5, Rab7, Rab9, Rab11, LAMP1 or LAMP2. Primary antibody binding was detected using appropriate Cy3-labeled secondary antibodies.

The glass bottom of the dishes was detached by immersing the underside in 750 ml Coverslip Removal Fluid (MatTek), after which cells were mounted onto microscopy slides with DAPI-Vectashield and sealed with nail varnish. Z-stacks were produced using a Zeiss LSM 510 Inverted microscope (63x objective, 216 µm pinhole). Post-imaging analysis was conducted in ImageJ (version 1.45, NIH, U.S.A.) by producing composite images from 5 stack slices (5 µm) and then analyzing the co-localization of granzyme B to the endosomal/lysosomal marker. For this, images were split into red and green channels using the ‘RG2B Colocalization’ plug-in and the overlap is seen as light areas in a converted binary image. For quantification, the ‘Histogram’ macro was used to measure size and intensity of light areas. Values were normalized to units per cell. (n = 3–6 for each staining).

### Tumor Spheroids

CT26 tumor cells (5×10^3^ in 200 µl RPMI 1640 medium) were pipetted into high affinity 96-well plates (Costar EIA/RIA) coated with 1% (v/v) sterile agarose (Sigma). Each initial seeding of 5×10^3^ cells into 96 well plates generated spheroids. On day 4, the spheroids were washed in PBS twice and transferred into agarose-free 96-well U-bottom low evaporation lid tissue culture plates (BD Biosciences). Single cell suspensions were derived from 10 tumor spheroids by incubating them for 15 min in trypsin/EDTA at 37°C. The Hsp70 status of tumor spheroids was comparable to that of monolayer CT26 tumor cells (60% Hsp70 membrane-positivity).

### Granzyme B (grB) and Camptothecin (cam) Treatment

Mouse cells and tumor spheroids were incubated with inactive and active human grB purified from mammalian HEK293 cells [18] and the topoisomerase I inhibitor cam at 37°C at the indicated concentrations.

### Colony Forming Assays (CFA)

CT26 tumor cells (1.5×10^2^) were plated in 24-well plates in 0.5 ml RPMI 1640 medium. The medium was exchanged after 24 h and CT26 cells were incubated with grB (0.04, 0.1, 0.2, 0.4, 0.6, 0.8, and 1 µg/ml in conditioned RPMI 1640 medium) for 7 days. After methanol fixation and crystal violet staining, colonies consisting of more than 50 cells were counted. The plating efficiency was determined as the number of counted colonies divided through the number of seeded cells and multiplied by 100. The survival fraction was calculated as number of treated cells divided by the number of untreated colonies.

### Caspase-3 Apoptosis Assay and Fluorescence Microscopy

Following treatment of CT26 tumor cells, tumor spheroids and normal cells (5×10^5^) with grB and cam, single cell suspensions were staining using an FITC-conjugated active caspase-3 antibody apoptosis kit according to the manufacturer’s instructions (BD Biosciences). Fluorescence analyses were performed using a FACSCalibur™ flow cytometer using equipped with CellQuest Pro (v6.0) acquisition and analysis software (BD Biosciences).

For immunofluorescence analysis, untreated or treated cells (0.25×10^6^ cells in 1.5 ml RPMI 1640 medium) were seeded into 2-well chamber slides (Thermo Fisher Scientific). After 48 h cells were washed in PBS and stained using a PE-conjugated active caspase-3 antibody. After another washing step, cells were covered in mounting medium containing DAPI (Vectashield; Vector Laboratories Inc., Burlingame, USA), visualized by light and fluorescence microscopy (Axioscop 2 plus/Axio Cam MRc5, Zeiss) and analyzed using AxioVision 4.7.1 (Zeiss) software.

### Measurement of Spheroid Diameters and Induction of Apoptosis in grB-treated Tumor Spheroids

Tumor spheroids were incubated with grB (0 to 80 µg/ml) or 4 µg/ml cam. Conditioned medium was exchanged every third day. The diameter of the tumor spheroids was regularly measured over 14 days by light microscopical analysis using an Axiovert 3500, 10x objective microscope and a FinePix S1 Pro; Carl Zeiss camera system (MicroImaging GmbH, Oberkochen, Germany).

### Treatment of Tumor-bearing Mice with Human grB

BALB/c mice were obtained from an animal breeding colony (Harlan Winkelmann) and maintained in pathogen-free, individually ventilated cages (Tecniplast). Animals were fed with sterilized, laboratory rodent diet (MEIKA) and used for experiments between 6 and 12 weeks of age. All animal experiments were approved by the “Regierung von Oberbayern” and were performed according to the institutional guidelines. For the tumor regression analysis, CT26 tumor spheroids were injected i.p. into mice on day 1. Tumor take was 60% in this model and only mice with ultrasound proven tumors were used for the experiments. All mice received equivalent-sized spheroids (500–600 µm) which had been generated from an initial *in vitro* seeding of 5×10^3^ tumor cells. Tumors grew at similar rates *in vivo* and this was confirmed by microscopic inspection after 7 days.

Active and inactive human grB (20 µg/g body weight) and PBS (ctrl) were injected i.v. on days 6, 7, 13, and 14 after tumor inoculation. Mice were sacrificed by cervical dislocation on day 21, at which time tumors and organs were collected. Overall survival was determined in CT26 tumor-bearing mice (tumor spheroids, i.p.; n = 15) that had been injected i.v. with active and inactive human grB (20 µg/g body weight).

### Intraoperative in vivo Imaging of Hsp70 Expression Using the cmHsp70.1 Monoclonal Antibody

CT26 tumor cells (1×10^6^, in 100 µl PBS) were subcutaneously injected into the neck area of 8–12 week old female BALB/c mice and 6 days thereafter, 100 µg Cy5.5-conjugated cmHsp70.1 mAb was injected (i.v.) into the tail vain. Twenty four hours later, mice were sacrificed and the fluorescence of the dissected tumor area was measured using an EM-CCD camera (iXon DV887, Andor, Belfast, Northern Ireland) equipped with a 710/10 nm band pass filter. For homogenous fluorophore excitation, laser light from a 450 mW 670 nm laser source (B&W tec. Inc., Newark, DE) was guided through a multimode fiber (200 lm core/0.22 NA) to a collimator and a diffuser (F260SMA-b; ED1-S20, Thorlabs, Newton, NJ). Sequential color images were taken from the identical area. Images were analyzed using imageJ processing and analysis software.

### Determination of Antibody Binding Capacity of Isolated CT26 Tumor Cells and Normal Endothelial Cells (ECs)

The capacity of CT26 cells that had been isolated from tumors and isolated endothelial cells to bind cmHsp70.1 mAb was determined using Quantum Simply Cellular anti-Mouse IgG beads according to the manufacturer’s protocol (Bangs Laboratories, Inc., Fishers, IN, U.S.A.). Briefly, beads with defined antibody binding capacity were incubated with cmHsp70.1-FITC mAb and analysed on a BD Biosciences FACSCalibur™ flow cytometer to obtain a standard curve. The fluorescence of cells was then measured using the same settings. The capacity of cells to bind cmHsp70.1-FITC is presented as mean equivalents of soluble fluorochrome (MESF) divided by the antibody binding capacity (ABC).

### Histochemistry and Immunohistochemistry

Formalin-fixed, paraffin-embedded tissues were serially cut (2.5 µm) and stained with eosin (eosin y-solution 0.5% aqueos, Merck) and hematoxylin (Mayer’s haematoxylin). Light microscopy images (10x objective) were recorded by Axioskop 2 plus Axio Cam MRc5 (Zeiss).

### Statistical Analysis

The significance of the data was determined by the Student’s t-test or the paired Student’s t-test using SigmaPlot (Erkrath, Germany). The significance levels were p* ≤ 0.05 (5%); p ** ≤ 0.01 (1%); p *** ≤ 0.001 (0.1%). Significant differences in overall survival were determined using the Generalized Wilcoxon test.
